# One-Pot Synthesis of Amphiphilic Linear and Hyperbranched Polyelectrolytes and Their Stimuli-Responsive Self-Assembly in Aqueous Solutions

**DOI:** 10.3390/polym17050701

**Published:** 2025-03-06

**Authors:** Angelica Maria Gerardos, Aleksander Forys, Barbara Trzebicka, Stergios Pispas

**Affiliations:** 1 Theoretical and Physical Chemistry Institute, National Hellenic Research Foundation, 48 Vassileos Constantinou Avenue, 11635 Athens, Greece; 2 Department of Chemistry, National and Kapodistrian University of Athens, Panepistimiopolis, Zografou, 15771 Athens, Greece; 3 Centre of Polymer and Carbon Materials, Polish Academy of Sciences, 34 ul. M. Curie-Skłodowskiej, 41-819 Zabrze, Poland; aforys@cmpw-pan.pl (A.F.); btrzebicka@cmpw-pan.pl (B.T.)

**Keywords:** amphiphilic copolymer, random copolymer, polyelectrolyte, hyperbranched

## Abstract

Stimuli-responsive polymeric nanostructures are compelling vectors for a wide range of application opportunities. The objective we sought was to broaden the array of self-assembling amphiphilic copolymers with stimuli-responsive characteristics by introducing a hydrophilic tunable monomer, (2-dimethylamino)ethyl methacrylate (DMAEMA), together with a hydrophilic one, lauryl methacrylate (LMA), within linear and branched copolymer topologies. Size exclusion chromatography was used to evaluate the resultant linear and hyperbranched copolymers’ molecular weight and dispersity, and FT-IR and ^1^H-NMR spectroscopy techniques were used to delineate their chemical structure. The structural changes in the obtained self-organized supramolecular structures were thoroughly investigated using aqueous media with varying pH and salinity by dynamic light scattering (DLS), fluorescence spectroscopy (FS), and transmission electron microscopy (TEM). The nanoscale assemblies formed by the amphiphiles indicate significant potential for applications within the field of nanotechnology.

## 1. Introduction

Stimuli-responsive (or “smart”) polymers represent a category of nanomaterials capable of undergoing reversible or irreversible alterations in their structure and physicochemical properties in response to various stimuli, both chemical and physical [1]. Numerous factors, including pH, temperature, and ion concentration, can trigger such changes. These environmental parameters can significantly influence the self-assembly processes in these materials [2]. Amphiphilic macromolecules, a significant class of such materials, are differentiated by the presence of both hydrophilic and hydrophobic moieties. When they are introduced into aqueous environments, this duality allows them to self-assemble into various nanostructures [3]. These macromolecules, akin to surfactants, create micelle-like structures, rendering them valuable for various applications, notably in nanomedicine, due to their capacity to facilitate hydrophobic compound encapsulation [4]. This process can be accomplished through non-covalent interactions such as hydrogen bonding and hydrophobic interactions. However, this approach often yields low encapsulation efficiencies, which may prematurely release the contents before they reach their intended environment in biomedical applications [5]. Macromolecules with modifiable properties are needed to overcome these limitations. Cationic macrosurfactants are given particular consideration among the amphiphilic compounds. Diverse cationic surfactants have been successfully developed, featuring various pendant groups, polymeric structures, etc. [6]. To achieve stimuli-responsive characteristics, the former is essential; amine-bearing polymeric structures are a desirable option because of their pH and temperature sensitivity. Striking examples are the (co)polymers based on poly[(2-dimethylamino)ethyl methacrylate] (PDMAEMA) and other alkylamine methacrylates, and poly(N-isopropylacrylamide). Moreover, the ability of DMAEMA’s amine group to form complexes through hydrogen bonding and electrostatic interactions makes it a highly suitable candidate for utilization as a functional monomer [7]. The pH-dependency of the tertiary amino group protonation characteristics of PDMAEMA dictates the moiety’s effective charge and, consequently, its water solubility. Additionally, it establishes the capacity to form complexes with anionic compounds, including biomolecules [8]. PDMAEMA exhibits thermoresponsiveness, whereby its lower critical solution temperature (LCST) is contingent upon its molecular weight and pH level. The presence of ionizable groups affects the LCST; specifically, it tends to decrease under basic conditions and increase in acidic environments. This phenomenon occurs due to the protonated state, which enhances the hydrophilicity of the polymer [9,10]. PDMAEMA-based copolymers have been effectively complexed with proteins [11], enzymes [12], and DNA [13]. Hydrophobic moieties are also important in producing structured nano-assemblies of amphiphiles. Bearing a long alkyl chain (n-dodecyl side chain), lauryl methacrylate (LMA) is a hydrophobic methacrylate ester-type monomer. The n-dodecyl chain provides ample side chain flexibility and high hydrophobicity compared to other alkyl methacrylates to facilitate spontaneous self-assembly. Moreover, LMA is appealing from an environmental standpoint and suitable for naturally produced copolymers as it can be made from plant oils, a renewable resource [14,15]. Hyperbranched polymers are gaining traction as a viable option for nanotechnology applications, leveraging the unique properties of copolymer topology. These materials are often more cost-effective than dendrimers while still providing similar functionalities, such as a high number of end and in-chain groups that can effectively interact and form complexes with various molecules [16]. Moreover, these polymers can be efficiently produced through a one-pot method. Established in 1998, reversible addition–fragmentation chain transfer (RAFT) polymerization has gained widespread recognition as a valuable one-pot technique for synthesizing complex macromolecules [17]. Its controlled polymerization characteristics make RAFT polymerization indispensable for developing sophisticated polymer topologies and multifunctional copolymers [18].

PDMAEMA has been successfully paired with various alkyl methacrylates, resulting in linear diblock [19,20] and triblock copolymers forming stimuli-responsive nanostructures in solutions. Our group has previously focused on the synthesis of the triblock copolymer PDEGMA-b-PDMAEMA-b-PLMA, which self-assembles in aqueous conditions to create spherical micellar structures. These structures consist of a stimuli-responsive PDMAEMA and PDEGMA corona surrounding a hydrophobic PLMA core [21]. In a similar fashion, PDMAEMA-b-PLMA-b-POEGMA amphiphilic triblock terpolymers have successfully encapsulated curcumin within spherical micelles [22]. Additionally, Jung et al. developed a triblock copolymer composed of hydrophilic PDMAEMA and PEG, which is end-capped by thermoresponsive tri(ethylene glycol) methyl ether methacrylate (TEGMA) units and hydrophobic n-butyl methacrylate (BuMA) moieties, resulting in multiresponsive micelles [23]. Similarly, Constantinou et al. synthesized terpolymers consisting of TEGMA, BuMA, and DMAEMA units resulting in spherical micelles [24]. Fan et al. synthesized ternary random copolymers that incorporate hydrophobic hexyl methacrylate moieties along with ionizable units of DMAEMA and MAA resulting in pH-sensitive micelles [25]. Non-linear copolymers are less researched. Skandalis et al. synthesized, utilizing the “arm first” method, amphiphilic mikto-arm PDMAEMA_x_PLMA_y_ stars [26]. In order to fully explore the potential of amphiphilic materials, targeted research should focus on simplified systems that effectively demonstrate their advantageous properties. Additionally, it is crucial to develop straightforward and efficient methods for synthesizing these novel polymer materials. By doing so, we can facilitate their scalability for a multitude of applications.

This work reports the synthesis of four amphiphilic random/statistical copolymers composed of DMAEMA and LMA, including two with a linear structure and two featuring a hyperbranched topology, utilizing RAFT polymerization. Research was conducted to assess the influence of pH variations and ionic strength on the behavior of the stimuli-sensitive polymeric nanostructures. This study is particularly significant as it explores, beyond the synthesis, the impact of macromolecular composition and non-linear, non-blocky architecture on the self-assembly process and the formation of amphiphilic copolymer nanostructures in aqueous media under different conditions.

## 2. Materials and Methods

### 2.1. Materials

DMAEMA, LMA, hydroquinone monomethyl ether (MEHQ) inhibitor remover, 2,2 azobisisobutyronitrile (AIBN), 4-cyano-4-(phenyl-carbonothioylthio)-pentanoic acid (CPAD), pyrene, and all solvents, including 1,4-dioxane (99.8% pure) and tetrahydrofuran (THF), were supplied by Sigma Aldrich (St. Louis, MO, USA). Ethylene glycol dimethacrylate (EGDMA) monomer, utilized as the branching agent, was purchased from Merck (Darmstadt, Germany). Water for injection (WFI) was purchased from DEMO AΒEΕ (Athens, Greece). The monomers were purified using a column packed with MEHQ inhibitor remover. Methanol was used to recrystallize AIBN. All the solvents used were of analytical grade.

### 2.2. Synthesis of Amphiphilic Copolymers

**Figure 1:**
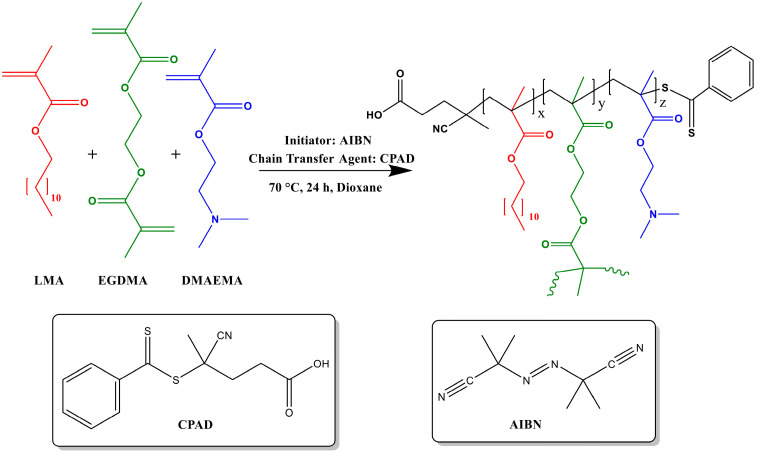
Synthesis route of hyperbranched H-P(LMA-co-DMAEMA) copolymer.

Linear P-(LMA-co-DMAEMA) was synthesized via RAFT polymerization (Figure 1). Briefly, AIBN (radical initiator), CPAD (chain transfer agent), 1,4-dioxane (reaction solvent), DMAEMA (hydrophilic monomer), and LMA (hydrophobic monomer) were added into a round-bottomed flask in appropriate quantities (see Table 1). The contents of the flask were then stirred vigorously utilizing a magnetic stirrer to ensure thorough mixing. Subsequently, the solution was de-aerated by bubbling with Ν_2_ for 20 min. Then the flask was set in an oil bath at 70 °C under magnetic stirring and the polymerization was allowed to proceed for 24 h. The mixture was then cooled to freezing temperature (~−20 °C) to quench the reaction. Finally, the frozen product was exposed to the ambient atmosphere to complete the polymerization process. The product was precipitated in a tenfold volume of cold hexane to remove unreacted monomers and other impurities. The precipitated polymer was separated by decantation, collected using THF, and dried in a vacuum oven for 72 h at an ambient temperature. The hyperbranched analogs were synthesized in the same manner (see Figure 1), with the exception of the addition of EGDMA as the branching agent in a 1.2:1 molar ratio to CPAD.

### 2.3. Colloidal Dispersion Preparation

Nanoprecipitation was selected as the aqueous polymer solution preparation technique because of its many benefits. The technique calls for two miscible solvents, one of which is typically water in excess. This technique’s fundamental concept involves the transition phase that occurs upon dispersion of the polymer solution in organic solvent into the aqueous medium. The polymer is dissolved in THF and afterwards, this solution is agitated and “violently” mixed/dispersed in an aqueous medium. The nanoparticles develop rapidly in an effort to evade the water molecules, thus forming nanodroplets in the aqueous phase. The organic solvent eventually evaporates with heat, creating an aqueous colloidal solution with newly formed nanoparticles [27]. Each copolymer was dissolved in THF (C_polymer_ = 5 × 10^−3^ g/mL). The solution was then injected into the appropriate volume of distilled water while being vigorously stirred at 55 °C. To ensure proper THF evaporation, heat was applied for a minimum of two hours before being left overnight at room temperature. Water was added as needed. The final copolymer concentration in every sample was 10^−3^ g/mL in 15 mL total volume of water.

### 2.4. Methods

#### 2.4.1. Size Exclusion Chromatography

Each copolymer’s molecular weight and molecular weight distribution were determined by employing a Waters SEC system. Three µ-Styragel mixed bed columns (with pore sizes varying from 10^2^ to 10^6^ Å), a Waters 1515 isocratic pump, and a Waters 2414 refractive index detector (equilibrated at 40 °C) constitute the system. The eluent, THF, was set at a flow rate of 1.0 mL/min and contained 5% triethylamine as a mobile phase additive. The average molecular weights of the linear monodisperse polystyrene standards used to calibrate the column set ranged from 1200 g mol^−1^ to 152,000 g mol^−1^. The data were collected and analyzed using the Waters Breeze software (Breeze v2.0, Waters Corporation, Milford, MA, USA).

#### 2.4.2. Proton Nuclear Magnetic Resonance Spectroscopy

Vnmrj software (VNMRJ 2.2C, Varian, Palo Alto, CA, USA) was utilized to acquire spectra using a Varian 300 (300 MHz) spectrometer. Both linear and hyperbranched polymers were dissolved in CDCl_3_ (C_polymer_ = 1–4 mg/mL). Parts per million (ppm) are used to express chemical shifts, with TMS acting as an internal reference. MestReNova software (MestReNova 14.0.0, Mestrelab Solutions, Bajo, Spain) was used to analyze the acquired spectra.

#### 2.4.3. Fourier Transform Infrared Spectroscopy

A Fourier transform instrument (Bruker Equinox 55, Bruker Optics GmbH, Ettlingen, Germany) equipped with a single-bounce attenuated total reflectance (ATR) diamond accessory (Dura-Samp1IR II by SensIR Technologies, Chapel Hill, NC, USA) was used to obtain mid-infrared spectra in the 500–4000 cm^−1^ region. On average, 100 scans were obtained with a resolution of 2 cm^−1^. A press was used to measure the solid polymers.

#### 2.4.4. Dynamic Light Scattering

An ALV/CGS-3 Compact Goniometer System (ALV GmbH, Hessen, Germany) was used to conduct DLS studies. The JDS Uniphase 22 mW He–Ne laser in this system operates at a wavelength of 632.8 nm. The goniometer was fixed at a 90° measurement angle. The system was connected to a digital ALV-5000/EPP multitau correlator with 288 channels. The cumulant method and the CONTIN algorithm were used to analyze the autocorrelation functions, averaging five measurements in 30 s duration each. Hydrophilic syringe PVDF filters (0.45 µm pore size) were used to filter all samples before conducting measurements.

#### 2.4.5. Electrophoretic Light Scattering

The results were obtained using a Nano ZetaSizer system (Malvern, Worcestershire, UK) equipped with a 4 mW He–Ne laser that operated at a wavelength of 633 nm and a scattering angle of 173°. The data established herein represent an average of 10 consecutive scans at ambient temperature. The Smoluchowski equation was used to analyze the collected data.

#### 2.4.6. UV–Vis Spectroscopy

A Perkin Elmer Lambda 19 UV–Vis–NIR spectrometer (Perkin Elmer, Waltham, MA, USA) was used to record the UV–Vis spectra in the 200–600 nm range. The scan speed was set to 240 nm/min for each measurement. Given the utilization of a double-beam spectrometer, a reference cuvette containing the dispersion medium served as the reference for all measurements.

#### 2.4.7. Fluorescence Spectroscopy

A Spectrofluorometer Fluorolog-3 Jobin Yvon-Spex (model GL3-21, Horiba, Kyoto, Japan) was used to record fluorescence spectra at ambient temperature throughout a wavelength range of 350–700 nm. The excitation wavelength was set at 335 nm, and the emission and excitation slits were both set at 2 nm in order to acquire pyrene spectra. The intensity of the first peak (I_1_) in the pyrene emission spectrum was divided by the intensity of the third peak (I_3_) to determine the I_1_/I_3_ ratio. A series of 11 copolymer concentrations were prepared with consecutive dilutions, with polymer concentrations ranging from 10^−3^ g mL^−1^ to 10^−8^ g mL^−1^. Each sample contained 0.1% *v*/*v* of a 1 mM pyrene solution in acetone (c_pyrene_ = 1 µM).

#### 2.4.8. Transmission Electron Microscopy

Transmission electron microscopy (TEM) images were obtained using a Tecnai F20 X TWIN microscope (FEI Company, Hillsboro, OR, USA) equipped with a field emission gun, operating at an acceleration voltage of 200 kV. Images were recorded on the Gatan Rio 16 CMOS 4 k camera (Gatan Inc., Pleasanton, CA, USA) and processed with Gatan Microscopy Suite (GMS) software version 3.5 (Gatan Inc., Pleasanton, CA, USA). For each measurement, 6 μL of solution was placed on a copper grid covered with carbon film and air dried at room temperature before conducting measurements.

## 3. Results and Discussion

### 3.1. Size Exclusion Chromatography Analysis

**Figure 2:**
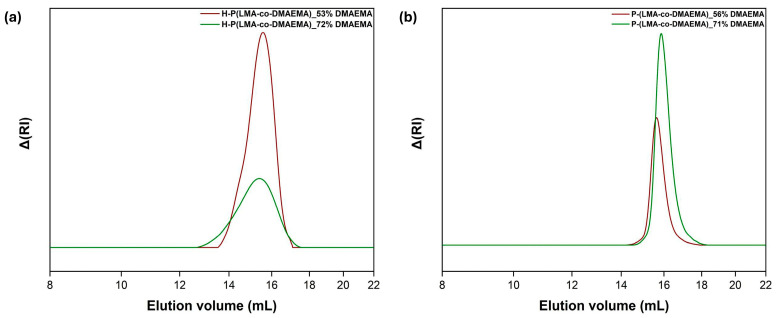
SEC curves of the hyperbranched (**a**) and linear (**b**) LMA/DMAEMA copolymers.

Figure 2 illustrates the synthesized copolymers’ SEC traces, validating each synthetic route’s success (see Table 1). Specifically, the linear copolymers produced a single sharp peak with a narrow distribution indicating unimodal polymer molecular weight distributions. On the contrary, the hyperbranched analogs generated monomodal molecular weight distributions with significantly larger polydispersity indexes. The SEC results show significantly higher weight-average molecular weights than stoichiometric calculations. This is due to the hyperbranched copolymer’s globular structure, which causes a significant difference in hydrodynamic volume compared to the linear standards used for calibration [28]. The dispersity values of the hyperbranched copolymers were higher than those of the linear analogs due to the branching process during polymerization.

### 3.2. Proton Nuclear Magnetic Resonance Spectroscopy Analysis

**Figure 3:**
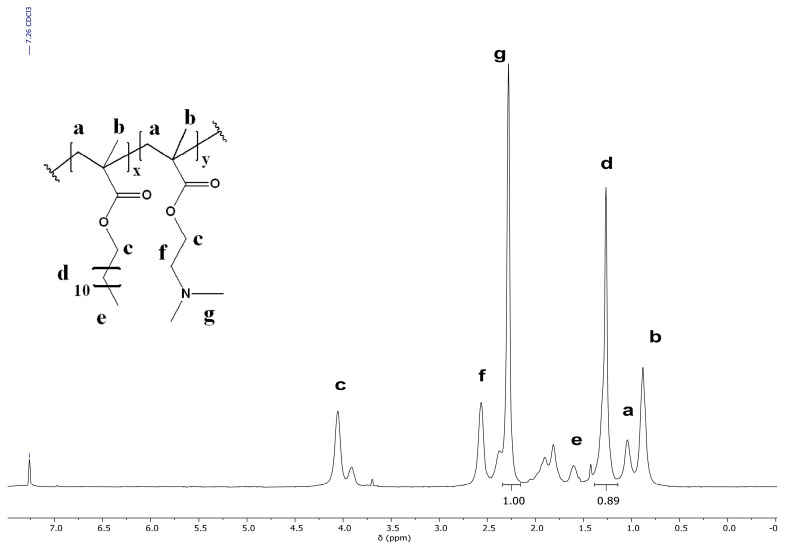
^1^H-NMR spectrum of P2 in CDCl_3_. Letters above the spectra peaks correspond to the relevant H nuclei shown in the chemical structure of the copolymer in the inset.

The chemical composition and structural identity of the aforementioned copolymers were determined qualitatively and quantitatively via proton NMR spectroscopy as shown in Figure 3. More specifically, stoichiometric compositions were calculated based on the major peaks on the NMR spectra, namely the -CH_3_ of the dimethylamino group of DMAEMA found at 2.28 ppm and the -CH_2_- of the LMA chain found at 1.27 ppm. The experimental compositions were estimated to be nearly identical to the targeted values. Yet, due to the similar chemical environment of the different hydrogen atoms present, quantitative analysis was notably challenging, a fact that justifies the deviation from the theoretical values.

### 3.3. Fourier Transform Infrared Spectroscopy Analysis

**Figure 4:**
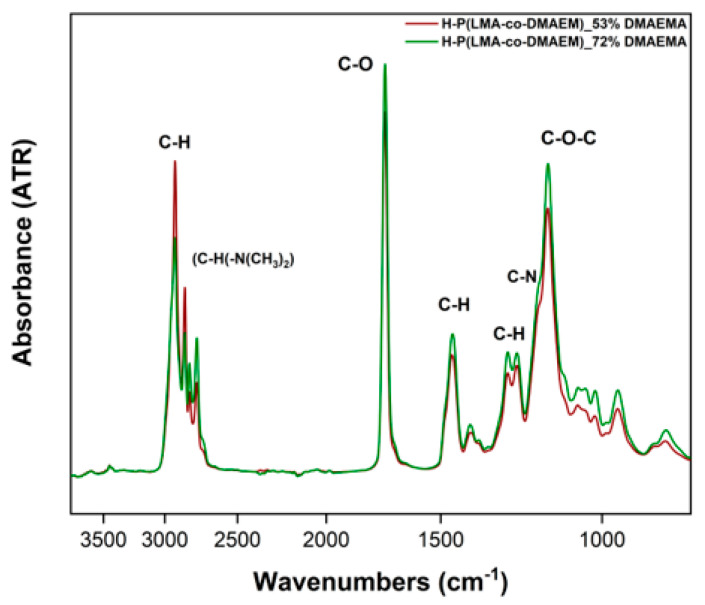
ATR-FTIR spectra of the hyperbranched copolymers (in the solid state).

FT-IR in conjunction with ^1^H-NMR spectra further confirm the chemical composition of the resulting copolymers. Beginning from the right, the primary peaks seen in Figure 4 were as follows: The C-O-C methacrylate stretch and the stretching vibration of the C-N bond in the DMAEMA unit correspond to the split in the 1145–1180 cm^−1^ range. The double intermediate peak (1240–1260 cm^−1^) can be attributed to C-H wagging or rocking vibration. C-H scissoring accounts for the 1460 cm^−1^ medium peak. The stretching vibration of C-O was linked to the sharp distinctive peak at 1765 cm^−1^. The characteristic bands of P(DMAEMA) are associated with (C-H(-N(CH_3_)_2_) stretching and are situated between 2820 and 2710 cm^−1^. The strong peak at 2925 cm^−1^ indicates asymmetric C-H stretching, while the neighboring peak represents symmetric stretching in the same group. Furthermore, the FT-IR spectra’s lack of a distinct C=C stretch band indicates that the monomers—including EGDMA vinyl units—are entirely consumed during polymerization [29,30].

### 3.4. Zeta Potential Analysis

In colloidal suspensions, zeta potential is a key parameter that influences biodistribution and the nanosuspensions’ stability. High zeta potential levels can enhance electrostatic repulsion forces between particles that help to maintain the stability of nanoformulations [31,32]. The existence of tertiary amine groups in the DMAEMA segments is responsible for the high positive values (see Table 2) observed throughout all samples. These suspensions are perfect for applications in nanomedicine since formulations with zeta potential values in this range (larger than a 30 mV absolute value) are regarded as pharmaceutically stable [33]. A high zeta potential value contributes to enhanced stability by reducing the likelihood of aggregation, thereby ensuring the effective delivery of therapeutic agents at the nanoscale. This stability is crucial for maintaining the efficacy and safety of nanomedicine formulations in various biomedical applications.

Based on the results, branching does not seem to affect zeta potential significantly. One should bear in mind that branched copolymers have a large number of -COOH groups originating from CTA fragments in the structure, while linear ones have only one -COOH group at the end of the chain. The protonation–deprotonation equilibria of carboxylic and amine chains and the degree of aggregation determine the overall charge of the structures in aqueous media. It seems that the higher number of amine groups originating from the DMAEMA segments dominate and determine in both cases the zeta potential values. In terms of the composition, the increase of DMAEMA from 54% to 70% in H1 and H2, respectively, induces a 24% decrease in zeta potential, which is unexpected. However, there is no hard evidence that could aid in ascribing this change to a specific phenomenon. Polar groups are expected to reside on the surface of the aggregates in contact with water, but the overall conformation of the associated chains within the aggregates determines the surface charge of the structures formed. Apparently, amphiphilic macromolecules arrange differently within the aggregates in the linear and branched copolymer cases.

### 3.5. Response to Solution pH Changes

**Figure 5:**
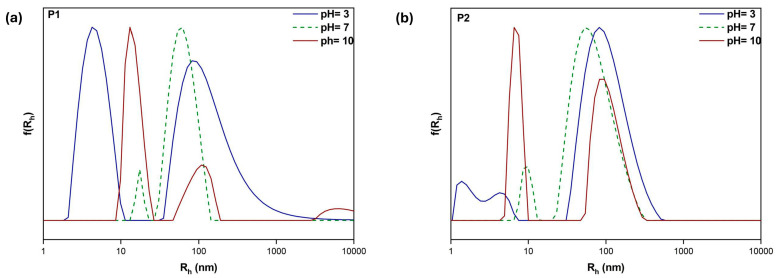
DLS size distributions for P1 (**a**) and P2 (**b**) linear copolymer aqueous solutions, C_polymer_ = 10^−3^ g/mL.

**Figure 6:**
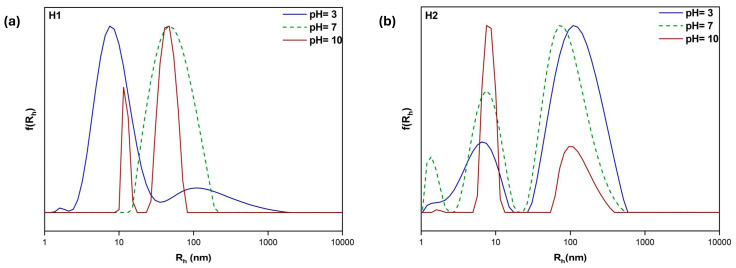
DLS size distributions for H1 (**a**) and H2 (**b**) hyperbranched copolymer aqueous solutions.

PDMAEMA is a weak cationic polyelectrolyte (pK_a_ = 7.5) which is largely water soluble at both neutral and acidic pH. The amine groups are partially protonated under these conditions, while the inverse is true at higher pH values [34]. This unique property makes PDMAEMA particularly suitable for drug delivery applications, as it leverages the lower acidity found in tumor tissues to facilitate sustained drug release [35]. To assess whether these characteristics were preserved in the copolymers, light scattering techniques were utilized at three distinct pH values: 3, 7, and 10 (refer to Figure 5 and Figure 6 and Table 3). These results allow for several conclusions. In most cases, two populations are observed, indicating the formation of distinct in size nanostructures through self-assembly, probably due to the random sequence of hydrophilic/hydrophobic segments in the copolymers. Aggregates with small and large numbers of polymer chains coexist. The linear copolymers are quite similar in terms of R_h_ in neutral conditions. In the case of H1, uniformly sized nanostructures with a rather large size dispersity and sufficient mass are formed at pH 7 while smaller-sized structures dominate at pH 3. Furthermore, there is a noticeable mass difference, as indicated by the scattered light intensities, between the 30–70 grouping (copolymers P2 and H2) and the 50–50 grouping (copolymers P1 and H1). This is explained by the fact that a higher percentage of hydrophobic LMA segments promotes more compact nanostructures with a higher mass, which raises the observed scattered light intensity values. Interestingly, hyperbranched and linear nanostructures differ in mass due to the former’s significantly higher number of -COOH polar end groups, which enhance the hydrophilicity of these systems and promote greater interactions with aqueous media. These structures noticeably rearrange when the pH is varied, as evidenced by the considerable variations in intensity, size, and PDI observed in each solution. Smaller populations are observed, particularly in acidic conditions (R_h_ = 1–9 nm), which in some cases can be attributed to non-aggregated copolymer chains present in the suspension. Additionally, a decrease in mass and an increase in size can be observed, which is associated with the PDMAEMA pendant groups’ higher charge density, which results in intense repulsion between the hydrophilic chains [36]. As more and more solvent accumulates in the nanostructures, this action causes swelling. Interestingly, hyperbranched and linear nanostructures differ in mass due to the former’s significantly higher number of -COOH polar end groups, which enhance the hydrophilicity of these systems at pH 10 and promote greater interactions with aqueous media. As the pH of the solution increases, a greater proportion of the carboxylic acid groups within the polymer become ionized. This ionization leads to enhanced electrostatic repulsion between the negatively charged carboxylate groups, which subsequently contributes to the expansion of the hydrophobic domains in the copolymer aggregates. At these pH levels, PDMAEMA’s hydrophobicity increases.

### 3.6. Behavior in Salt Solutions

**Figure 7:**
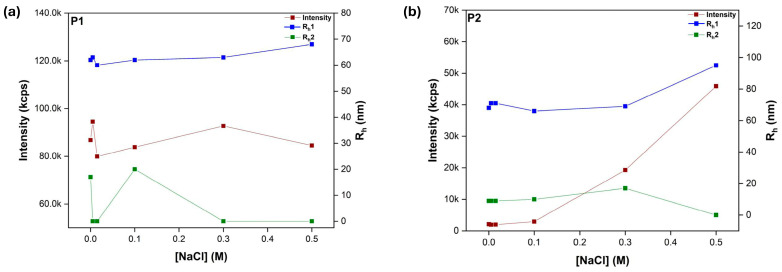
Collective DLS data as a function of salinity. (**a**) P1 and (**b**) P2 linear copolymers, C_polymer_ = 10^−3^ g/mL, pH = 7.

**Figure 8:**
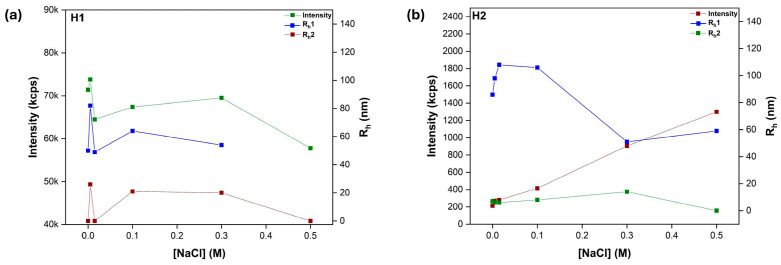
Collective DLS data as a function of salinity. (**a**) H1 and (**b**) H2 hyperbranched copolymers, C_polymer_ = 10^−3^ g/mL, pH = 7.

Amphiphilic polyelectrolyte self-assembly is affected by ionic strength, as the addition of salt alters the strength of the electrostatic interactions between charged moieties. Specifically, higher ionic strength typically screens these electrostatic interactions, making them weaker and allowing for greater rearrangement or aggregation of the polyelectrolyte chains. Therefore, amphiphilic ionic copolymers should exhibit variable structural characteristics in response to fluctuations in the solution’s ionic strength [37]. Τopology seems to produce small effects, as copolymer compositional analogs produced similar behavioral motifs. The intensity values in the 50–50 composition grouping (copolymers P2 and H2 see Figure 7 and Figure 8) increase and then quickly decline with salt concentration. This could be explained as the structures “tightening up” due to an overabundance of ions, which causes swelling. These structures appear to stabilize following the salt concentration increase. Regarding the other group, a mass increase is noted, which may be explained by the fact that hydrophobic interactions are the primary driving force behind self-assembly, which results in more compact structures.

### 3.7. Microenvironment Polarity Through Pyrene Fluorescence

**Figure 9:**
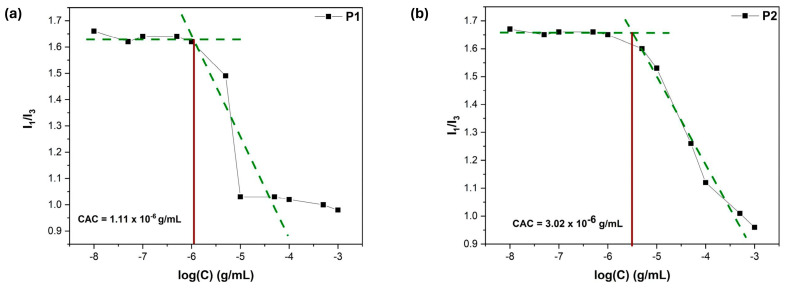
I_1_/I_3_ vs. polymer concentration plots for CAC determination regarding (**a**) P1 and (**b**) P2 copolymer aqueous solutions, C_pyrene_ = 1 μM. (Green lines refer to the two tangent lines described in the text).

**Figure 10:**
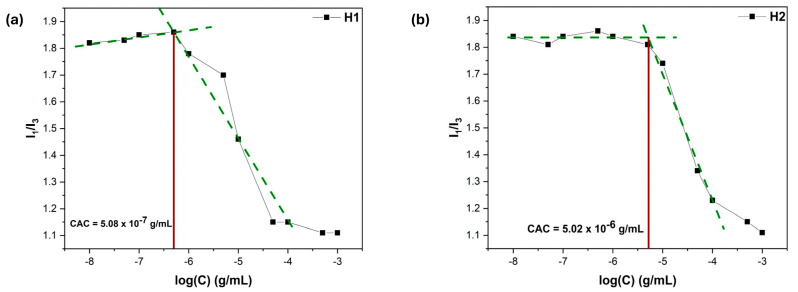
I_1_/I_3_ vs. polymer concentration plots for CAC determination regarding (**a**) H1 and (**b**) H2 copolymer aqueous solutions. (Green lines refer to the two tangent lines described in the text).

Pyrene has been used as a fluorescent probe because it can provide a thorough understanding of amphiphilic copolymer self-assembled nanostructures in aqueous conditions [38]. In addition to serving as a sufficient measure of hydrophobicity, the I_1_/I_3_ value is often utilized to determine the critical aggregation concentration (CAC) values. This involves plotting the I_1_/I_3_ value against the logarithm of the polymer concentration. Specifically, the CAC is defined as the point where two tangent lines intersect over the steep decline observed in the I_1_/I_3_ value as the concentration of the copolymer increases [39]. As expected, H1 and P1 present lower CAC values, as the higher LMA content adds to the hydrophobicity of the nanostructures (see Figure 9 and Figure 10). This improved stability can be linked to the stronger interactions among the hydrophobic regions in the nanostructures, resulting in more robust structural integrity and allowing them to maintain their form upon dilution in high volumes. As anticipated, the comparison of I_1_/I_3_ across varying pH levels reveals a distinct trend: higher values are observed in acidic conditions, while lower values are found in basic conditions (see Table 4). This indicates that the hydrophobicity of the nanostructures changes significantly depending on the acidity or alkalinity of the environment, just as observed in the DLS measurements.

### 3.8. Transmission Electron Microscopy Imaging

**Figure 11:**
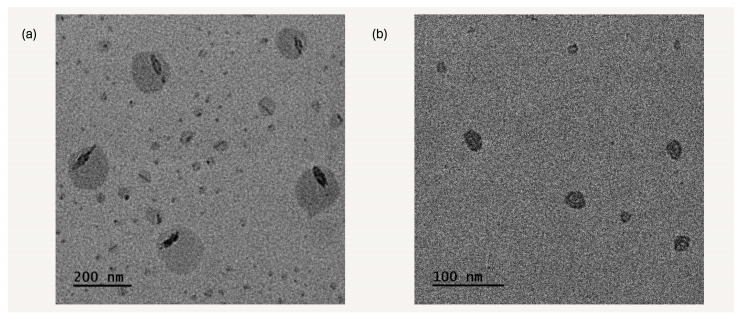
TEM images from (**a**) P1 and (**b**) P2 copolymer solutions.

Transmission electron microscopy (TEM) is recognized as a powerful imaging tool for studying the structural features of copolymer nanostructures. This technique allows for precise observation of the morphology of polymer nanostructures and the interfaces between different inner domains [40]. In the P1 solutions, particles of both spherical and irregular shape were identified, with noticeable high-contrast rod-like structures present throughout (see Figure 11). These rod-like structures are likely LMA domains, which exhibit a higher contrast due to their increased compactness/hydrophobicity compared to the hydrophilic, water swollen, outer DMAEMA domains in the self-assembled structures. The particle sizes range from 10 to 220 nm, with an average size of 50 nm based on the image analysis of 200 objects.

Particles in the P2 solutions exhibit both spherical and irregular shapes, with sizes ranging from 50 to 80 nm. Some particles are larger, measuring between 150 and 200 nm, while smaller particles, approximately 5 to 15 nm, can be found on their surfaces. Smaller spherical aggregates seem to cluster together to form larger structures, while the lower contrast gaps observed in larger particles can be assigned to the DMAEMA nanodomains swollen by water molecules.

Regarding H1, particles are mainly characterized by a spherical shape. Moreover, small spherical particles resembling those in the P2 sample, with diameters ranging from 5 to 15 nm, have also been identified. Just as in the case of P2, DMAEMA nanodomain gaps are again observed.

**Figure 12:**
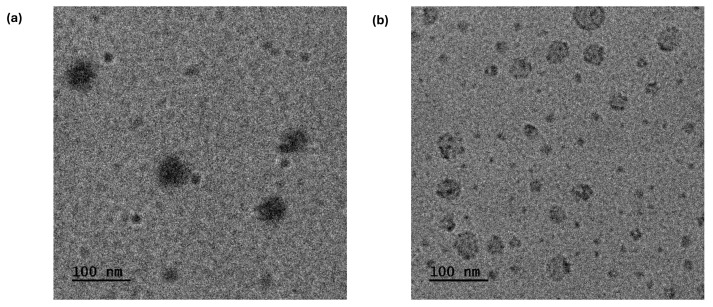
TEM images from (**a**) H1 and (**b**) H2 copolymer solutions.

Particles exhibiting both spherical and irregular shapes were identified in the H2 copolymer solutions, with a lower portion of their surfaces displaying enhanced contrast. Such high contrast domains are randomly distributed within the particle area and may be rich in LMA segments. The average size of these particles, determined from the measurement of 200 objects, was approximately 27 nm. The correlation between this topology and the nanostructures is emphasized by the formation of seemingly vesicular structures in the H2 hyperbranched copolymers (e.g., Figure 12b upper part). Moreover, similar to the findings in P2, small spherical high-contrast domains were observed on the surfaces of larger particles, measuring between 5 and 15 nm in size. Overall, the large variety of nanostructures observed in the copolymer solutions should be attributed to the statistical arrangement of hydrophobic segments within the macromolecules, as well as to the existing compositional heterogeneity of the amphiphilic copolymers.

## 4. Conclusions

To summarize, the findings of this work contribute to the general knowledge regarding the synthesis of linear and hyperbranched amphiphilic polyelectrolyte-type copolymers and their self-assembly behavior through the formation of nanostructures in solutions. The pH and salt-responsive properties of the PDMAEMA component together with variations in copolymer composition allow for fine-tuning of the structure of the formed nanoparticles. The nanoparticulate systems formulated and characterized exhibited a high surface charge, which renders them colloidally stable and allows them to interact with anionic species (including nucleic acids and proteins). Their amphiphilicity allowed for the encapsulation of pyrene, which gave further information about the micropolarity of the microenvironment within the nanoparticles and its variation with the solution pH, indicating their medicinal application as nanocarriers of hydrophobic compounds. Such systems could be engineered for the transfer and targeted release of their functional payload in acidic cancerous cell environments. Finally, TEM imaging provided insight into the footprint of such nanostructure collections and emphasized the impact of comonomer statistical distribution within the macromolecular chains as well as their global topology.

## Figures and Tables

**Table 1:** Polymer synthesis and characterization data.

Polymer	Code	mmol Ratios ^a^	M_w_ (g/mol) (×10^4^) ^b^	M_w_/M_n_ ^b^	%wt DMAEMA ^c^
P-(LMA-co-DMAEMA)	P1	3.9:6.4:0.2:0.1	9.7	1.17	56
P-(LMA-co-DMAEMA)	P2	2.4:8.9:0.2:0.1	7.6	1.2	70
H-P(LMA-co-DMAEMA)	H1	3.9:6.4:0.24:0.1:0.2	14.9	1.4	54
H-P(LMA-co-DMAEMA)	H2	2.4:8.9:0.24:0.1:0.2	20.1	1.9	70

^a^ LMA:DMAEMA:CPAD:AIBN:EGDMA; ^b^ determined by SEC; ^c^ determined by ^1^H-NMR. P denotes linear copolymer and H hyperbranched copolymer.

**Table 2:** ζ-potential values of copolymer solutions at pH = 7, C_polymer_ = 10^−3^ g/mL.

Sample	ζ-Potential (mV)
P1	+49
P2	+44
H1	+50
H2	+38

**Table 3:** DLS data emphasizing pH variations in the formed copolymer nanostructures, C_polymer_= 10^−3^ g/mL.

Sample	pH	Intensity (kcps)	PDI	R_h_ (nm)
P1	3	282	0.472	4 133
	7	86,800	0.257	17 62
	10	3064	0.426	14 100
P2	3	119	0.558	1 4 98
	7	2091	0.391	9 68
	10	816	0.519	7 106
H1	3	195	0.401	9 108
	7	71,400	0.249	50
	10	1409	0.354	12 45
H2	3	198	0.547	6 122
	7	212	0.515	1 7 89
	10	607	0.473	8 122

**Table 4:** Pyrene fluorescence data in copolymer aqueous solutions, C_polymer_ = 10^−3^ g/mL, C_pyrene_ =1 μM.

Sample	pH	I_1_/I_3_
P1	3	1.29
	7	0.98
	10	1.11
P2	3	1.43
	7	0.96
	10	1.07
H1	3	1.26
	7	1.11
	10	1.10
H2	3	1.44
	7	1.11
	10	1.17

## Data Availability

Data produced in this study are included in the manuscript and the Supplementary Materials.

## Supplementary Materials

The following supporting information can be downloaded at: https://www.mdpi.com/article/10.3390/polym17050701/s1. Figure S1. ATR-FT-IR spectra of the neat linear copolymers; Figure S2. ^1^H-NMR spectrum of P1 in CDCl_3_; Figure S3. ^1^H-NMR spectrum of H1 in CDCl_3_; Figure S4. ^1^H-NMR spectrum of H2 in CDCl_3_.
