# Supplementary material for: One-Pot Synthesis of Amphiphilic Linear and Hyperbranched Polyelectrolytes and Their Stimuli-Responsive Self-Assembly in Aqueous Solutions
Source: Polymers (Basel). 2025 Mar 6;17(5):701. doi: 10.3390/polym17050701 (PMC11902553; doi:10.3390/polym17050701)
Supplement: Supplementary file 1 [file polymers-17-00701-s001.zip › polymers-3480847-supplementary.pdf]

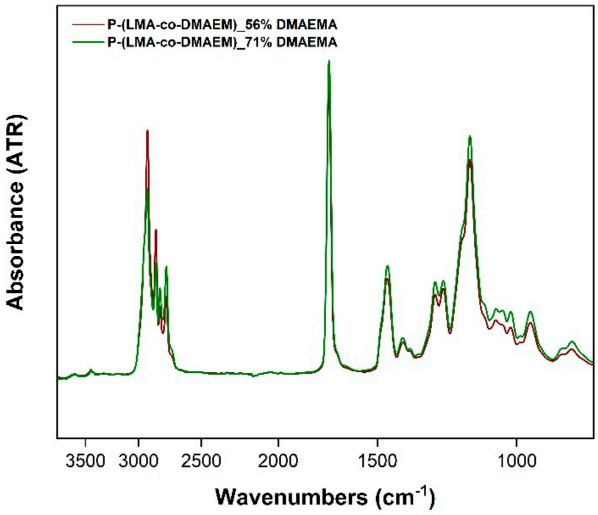

Figure S1. ATR-FT-IR spectra of the neat linear copolymers.

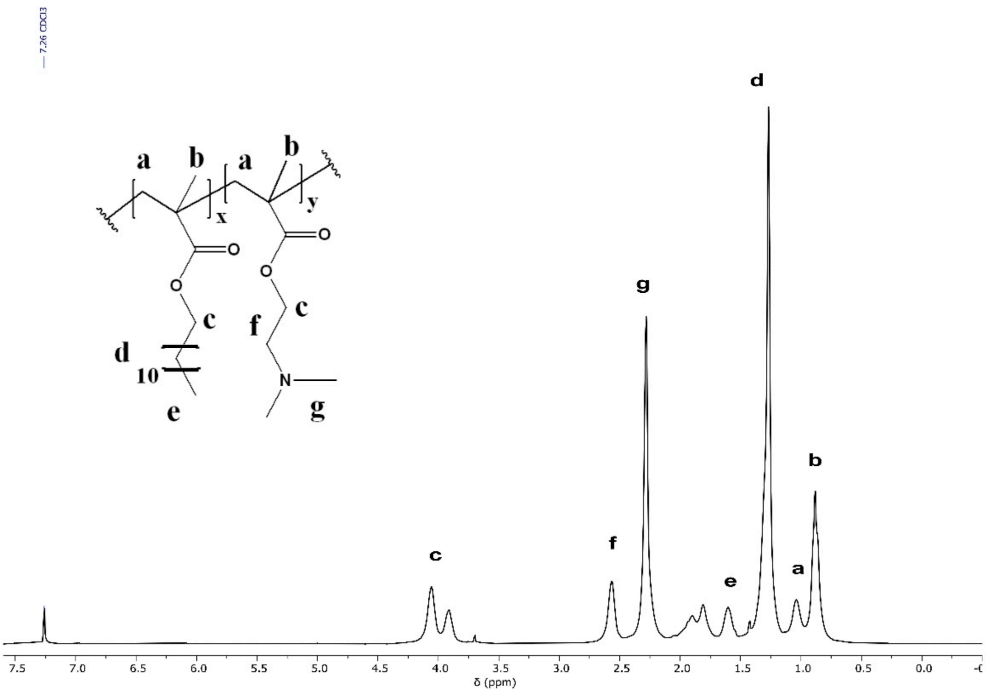

Figure S2. ^{1}H- NMR spectrum of P1 in $CDCl_3$.

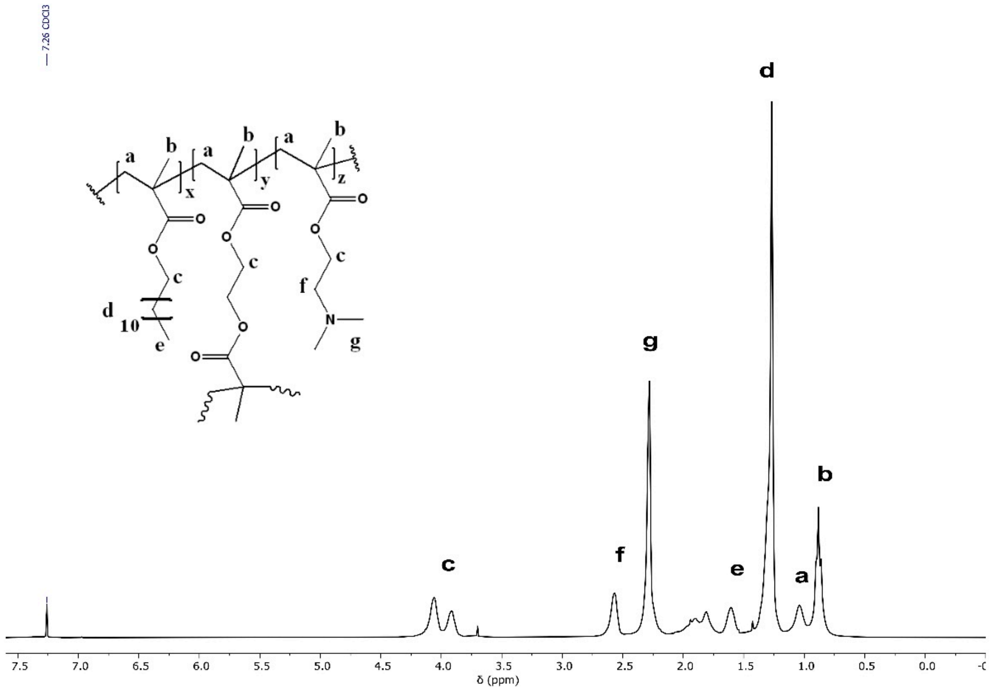

Figure S3. ^{1}H- NMR spectrum of H1 in $CDCl_3$.

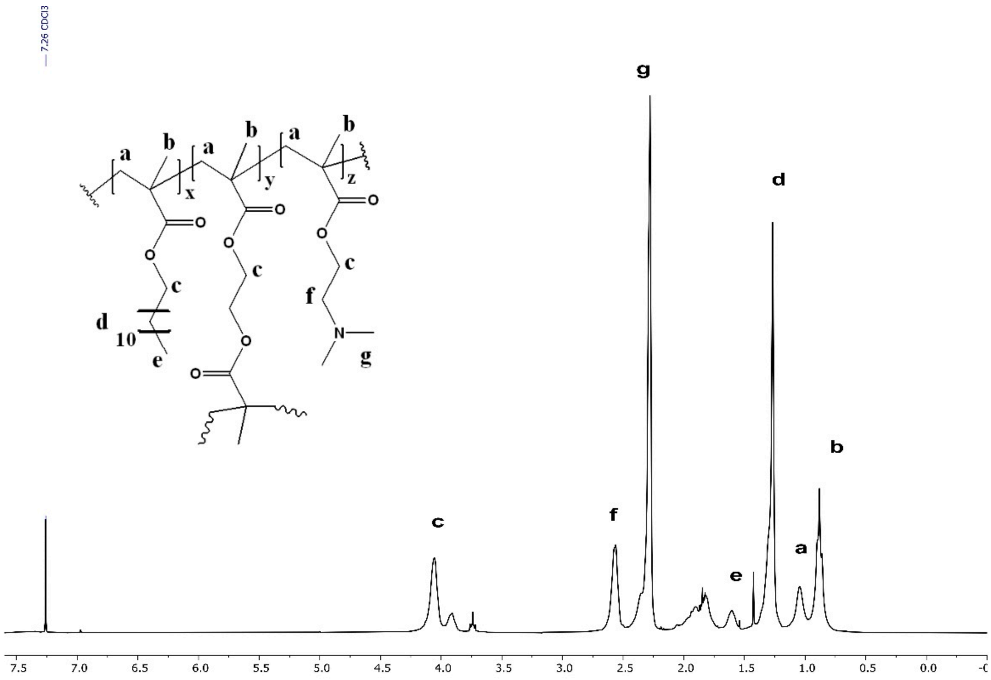

Figure S4. ^{1}H- NMR spectrum of H2 in $CDCl_3$.
